# Limited Microbial Contribution in Salt Lake Sediment and Water to Each Other’s Microbial Communities

**DOI:** 10.3390/microorganisms12122534

**Published:** 2024-12-09

**Authors:** Mingxian Han, Huiying Yu, Jianrong Huang, Chuanxu Wang, Xin Li, Xiaodong Wang, Liu Xu, Jingjing Zhao, Hongchen Jiang

**Affiliations:** 1 State Key Laboratory of Biogeology and Environmental Geology, China University of Geosciences, Wuhan 430074, China; hanmingxian@cug.edu.cn (M.H.); huangjianrong@cug.edu.cn (J.H.); shaodong@cug.edu.cn (X.W.); xuliuu@cug.edu.cn (L.X.); zhaojingjing@cug.edu.cn (J.Z.); 2 Shanxi Key Laboratory of Yuncheng Salt Lake Ecological Protection and Resource Utilization, College of Life Sciences, Yuncheng University, Yuncheng 044000, China; wangchuanxu@ycu.edu.cn (C.W.); lixin1981@ycu.edu.cn (X.L.)

**Keywords:** source tracker, microbial community, sediment and water, metabolic function, saline lakes

## Abstract

Climate change and human activities have led to frequent exchanges of sedimentary and aquatic microorganisms in lakes. However, the ability of these microorganisms to survive in their respective habitats between saline lake sediment and water remains unclear. In this study, we investigated microbial sources and community composition and metabolic functions in sediments and water in Yuncheng Salt Lake using a combination of source tracking and Illumina MiSeq sequencing. The results showed that 0.10–8.47% of the microbial communities in the sediment came from the corresponding water bodies, while 0.12–10.78% of the sedimentary microorganisms contributed to the aquatic microbial populations, and the microbial contributions depended on the salinity difference between sediment and water. Habitat heterogeneity and salinity variations led to the differences in microbial diversity, community composition, and assembly between sediment and water communities. The assembly of sedimentary communities was mainly controlled by stochastic processes (>59%), whereas the assembly of aquatic communities was mainly controlled by deterministic processes (>88%). Furthermore, sediments had a higher potential for metabolic pathways related to specific biogeochemical functions than lake water. These results provide insights into the survival ability of microorganisms and the mechanisms of microbial community assembly under frequent exchange conditions in saline lakes.

## 1. Introduction

Sediments and overlying water are the two main habitats of lake ecosystems, representing distinct habitats, both of which contain enormous microbial diversity. Surface sediments are in constant contact with the water column above and receive nutrients from it [1,2]. Climate change and human activities are the driving forces of microbial diversity [3]. Climate change-related phenomena such as rising temperatures, salinity variations, and changing precipitation patterns, as well as human activities such as pollution and land-use changes, could lead to increasingly frequent disturbances between sediments and overlying water columns [4], resulting in increased microbial exchange between the two habitats. However, our understanding of the ability of exchanged microorganisms to survive in these two contrasting environments (sediment and aquatic habitats) remains limited.

Sediments and aquatic habitats exhibit significant differences in physical and chemical properties, resulting in distinct biogeochemical patterns that drive the development of microbial communities in different ways in these unique environments [5]. Previous studies have demonstrated significant differences in microbial communities living in freshwater lake water and sediments [6,7]. In addition, microorganisms play an important role in biogeochemical processes in both water and sediments. For example, putative functions such as aerobic chemoheterotrophy and phototrophy were more common in lake water; in lake sediments, the degradation of biomass, aromatic compounds, and hydrocarbons, as well as the transformation of metals and sulfur, were mainly observed [6]. Another study showed significant differences in extracellular hydrolytic enzyme activity between seawater and sediments in marine environments [8]. However, most existing studies have mainly focused on the microbial community composition and metabolic functions in sediments and water with salinity up to 35 g/L (salinity of seawater). Currently, our knowledge of microbial distribution patterns and biogeochemical functions is limited between sediment and water columns in saline lakes (with salinity above that of seawater) [9,10]. Saline lakes are widespread worldwide and account for almost half of the total area of all lakes on Earth [11,12]. Therefore, studying the differences in microbial community structures and metabolic functions between sediment and water of saline lakes are crucial to improve our understanding of microbial ecology in lake ecosystems.

Elucidating the underlying mechanisms of microbial community assembly remains a central topic in microbial ecology [13]. It is currently widely accepted that both deterministic and stochastic processes play a role in the assembly of microbial communities [14,15,16]. Although our understanding of microbial community assembly has made significant progress in the last decade, we have still not reached a clear consensus on the relative importance of deterministic and stochastic processes in shaping microbial structure [13,16,17]. For example, some studies indicate that stochastic processes appear to dominate the assembly mechanisms of microbial communities in lake water [18,19]. On the contrary, other studies suggest that aquatic bacterial communities were mainly influenced by deterministic processes closely linked to their habitats [20]. Likewise, stochastic processes are more important than deterministic processes in the formation of bacterial antibiotic communities in lake sediments [21]. Another study found that deterministic processes outperformed stochastic influences in controlling the assembly of benthic bacterial communities [7]. These contradictory results suggest the need to further evaluate how the relative importance of deterministic and stochastic processes shapes microbial communities in sediments and water. Sediments (non-fluidic) and water (fluidic) form two different habitats in lake ecosystems [10]. Previous studies have shown that system fluidity can influence the relative contributions of deterministic and stochastic processes to community assembly due to diffusion limitations [22]. However, the relative importance of deterministic and stochastic processes for the shaping of microbial communities between sediments (non-fluidic) and water (fluidic) in saline lakes is not yet fully understood.

Given the significant difference in physiochemical properties between sediment and water habitats, we hypothesize that (i) in saline lakes, sedimentary microbial communities may not be able to survive in the overlying water, and vice versa; (ii) aquatic and sedimentary microbial communities can have different community compositions and assembly mechanisms; (iii) sediments are expected to have a higher potential for specific biogeochemical processes. However, there is limited knowledge about the ability of these microorganisms to survive in their respective habitats as well as the differences in microbial community composition and metabolic functions between saline lake sediment and water. To address the above knowledge gaps, we investigated sources and the composition and functions of microbial communities involved in biogeochemical cycles in sediments and water in the Yuncheng Salt Lake using a combination of source tracking and Illumina MiSeq sequencing. The objectives of this study are: (1) to evaluate the microbial contribution in salt lake sediments and water to each other’s microbial communities; (2) to quantify the relative contributions of deterministic and stochastic processes in shaping aquatic and sedimentary communities; (3) to determine the difference in microbial community composition and potential metabolic functions between lake sediments and water.

## 2. Materials and Methods

### 2.1. Study Sites

Yuncheng Salt Lake, also called the “Dead Sea of China”, is located in the southern suburbs of Yuncheng City in Shanxi Province in eastern China and covers an area of 120 km^2^ [23]. It is the third largest natural inland lake of sodium-sulfate (Na_2_SO_4_) type in the world [24]. Yuncheng Salt Lake is not a monolithic body of water; rather, it is divided by numerous dams into various salt pans of different sizes and varying salinity. As the most important saltwater lake, it is known for its rich mineral resources and unique halophilic algae. The study area is a semi-closed intermontane basin with mountains to the south, east, and north [25]. Under East Asian monsoon climate conditions, the annual average precipitation is 550 mm/year, while the total annual evapotranspiration is more than 2000 mm [26].

### 2.2. Sample Collection and Physicochemical Measurements

The sampling and on-site measurements were carried out in the Yuncheng Salt Lake in mid-September 2023. During the sampling campaign, a total of 30 samples (15 sediment samples and 15 water samples) were collected from five locations (Figure S1). The five sites were distributed on the eastern side of the Yuncheng Salt Lake, where they have representative salinities and together form a salinity range. The geographical locations of the sampling sites were determined using a portable GPS unit, eTrex H (Garmin, Olathe, KS, USA). Surface sediments (approximately 1 to 5 cm deep) were collected from each sampling site in triplicate at each sample site using a sterile sampling spatula, while water samples (above the surface sediment) were collected in triplicate using a water sampler. Before use, the collection devices were cleaned and rinsed with alcohol and ultrapure water, respectively. Sediment samples intended for microbial DNA extraction and physicochemical measurements were transferred to sterile Ziploc bags, stored in refrigerated containers, and immediately transported to the laboratory. For aquatic microbial community analyses, approximately 500 mL of water samples were filtered through 0.22 μm diameter filter membranes (Whatman, Buckinghamshire, UK), with biomass-containing membranes collected aseptically into sterilized tubes and stored at −80 °C until genomic DNA extraction. Approximately 500 mL of lake surface water was collected in autoclaved polycarbonate bottles (Nalgene, Rochester, NY, USA) for subsequent physicochemical analysis.

In the laboratory, sediment porewater was obtained by centrifugation at 12,000× *g* for 10 min. The salinity and pH of sediment porewater and lake water were determined using a portable water multiparameter device (SANXIN, Shenyang, China). Major cation and anion concentrations, including K^+^, Na^+^, Mg^2+^, Ca^2+^, Cl^−^, and SO_4_^2−^, in both lake water and sediment porewater were measured using Dionex DX-600 ion chromatography (Dionex, Sunnyvale, CA, USA).

### 2.3. DNA Extraction and Illumina Sequencing

Extraction and purification of total DNA from 0.5 g of sediment and filter membranes was carried out using the Fast DNA SPIN Kit (MP Biomedical, Santa Ana, CA, USA) according to the manufacturer’s instructions. 16S rRNA genes of the extracted DNA samples were amplified using the universal primer set 515F (5′-GTGYCAGCMGCCGCGGTAA-3′)/806R (5′-GGACTACNVGGGTWTCTAAT-3′) as previously described [27]. The sequencing adapter and forward primer were linked by a unique 12 bp barcode to identify different samples. Gene amplification was performed in a 50 μL reaction system. PCR parameters were 94 °C for 3 min, followed by 35 cycles of denaturation at 94 °C for 45 s, annealing at 50 °C for 60 s, extension at 72 °C for 90 s; with a final extension at 72 °C for 10 min [27]. For each sample, triplicate amplifications were carried out, and the PCR products (~400 bp) were purified using a DNA Gel Extraction Kit (Axygen, Union City, CA, USA). Subsequently, the purified PCR products were pooled at equimolar concentrations and then sequenced on the Illumina Miseq platform (paired-end sequencing of 2 × 250 bp).

Processing of raw 16S rRNA sequencing data was performed in the QIIME2 program (version 2023.2) according to recommended protocols [28]. Briefly, sequence demultiplexing, joining, filtering, denoising, and chimera checking were conducted using the default plugins in QIIME2. Reads containing ambiguous bases or mismatches with the barcode or primers were removed, and feature tables were generated by using the DADA2 method [29]. Amplicon sequence variants (ASVs) (at the 100% identity level) [30] were reported as marker genes in this study. The taxonomy of representative ASV sequences was assigned using the SILVA 138 database with a confidence cutoff of 80%. Non-bacterial and chloroplast-associated ASVs were excluded for subsequent analysis. Finally, the resulting feature tables were rarified to equal sequence numbers (n = 56,425) before downstream analyses. All sequences used in this study are publicly accessible in the NCBI Sequence Read Archive (SRA) database under accession number PRJNA1161028.

### 2.4. Microbial Source Tracker

Source Tracker is a promising method for identifying the origins of communities and populations and has been widely used in previous studies [31,32]. Fast expectation-maximization microbial source tracking (FEAST) analysis using an expectation-maximization algorithm provides estimates to distinguish different source types while reporting the proportion contributed of unknown sources [33]. In this study, aquatic microbial communities in the lake were considered as a source and sediment communities as a sink to investigate the contribution of microorganisms in lake water to sediments. Similarly, when studying the contributions of sediment microorganisms to lake water, sedimentary microbial communities were selected as the source and aquatic communities as the sink. The input file for FEAST analysis was prepared following the guidelines of the FEAST package using 1000 iterations.

### 2.5. Statistical Analysis

Various packages in the R program were used for statistical analysis. The α-diversity indices (observed ASVs and Shannon index) were calculated using the ‘vegan’ package and visualized using the “ggpubr” package. The Wilcox test was employed to detect significant differences in the observed ASVs and Shannon indices between sediment and water samples. Principal coordinate analysis (PCoA) and Adonis tests (Bray-Curtis distance, permutations = 999) were conducted to examine differences in community composition between sediment and water samples using the “microeco” package. To identify highly different taxonomies (known as “biomarkers”) between sediment and water samples, the linear discriminant analysis (LDA) effect size (LEfSe) from the “microeco” package was used, and set the filter value of the LDA score was set to 4.0. Mantel tests were performed to examine the possible relationship between environmental factors and the Hellinger-transformed microbial community dissimilarities (Bray–Curtis distances with 9999 permutations) using the “vegan” package. Spearman’s rank correlations were performed to assess the correlation for each pair of environmental variables using the “Hmisc” package. Null model analysis (999 randomizations) was carried out to quantify the ecological processes influencing both the sediment and aquatic microbial community assembly [34]. Ecological processes are categorized into deterministic processes (homogeneous and variable selections) and stochastic processes (dispersal limitation, homogenizing dispersal, and non-dominant) [13,17]. Detailed quantification of the microbial community assembly process was described in our previous studies [18,35].

Phylogenetic Investigation of Communities by Reconstruction of Unobserved States (PICRUSt2; https://github.com/picrust/picrust2, accessed on 1 March 2024)) was adopted to predict the functional potential of the microbial community based on the relative abundance of marker gene sequences in samples [36]. This approach allowed inference of the relative abundance of potential metabolic pathways from the 16S rRNA gene sequence profiles. The abundance of MetaCyc pathways was calculated in PICRUSt2 using structured mappings of Enzyme Commission gene families to pathways [37]. A differential abundance analysis of predictive functional pathways was performed using the pathway_daa function using the LinDA method in the “ggpicrust2” package [38]. Heatmap analysis was then performed for the abundance of MetaCyc pathways and differentiations using principal component analysis between sediment and water samples. Annotation and quantification of microbial functional genes involved in the microbially mediated C/N/S geochemical cycle processes (based on the DiTing pipeline) occurring in sediment and water samples were performed on the Wekemo Bioincloud interactive platform [39].

## 3. Results

### 3.1. Physiochemical Characteristics in the Sediment and Water Samples

The physicochemical parameters of the sediment samples showed significant differences from those of the lake water (Table S1). For example, the salinity of the sediment pore water ranged from 55.20 g/L to 125.20 g/L, which was lower than that of the lake water (ranging from 96.20 g/L to 296.40 g/L). Accordingly, the concentrations of the major ions (e.g., K^+^, Na^+^, Mg^2+^, Cl^−^, and SO_4_^2−^) were higher in the lake water than in the pore water of the lake sediment. The pH values in both the sediment pore water and the lake water were alkaline (pH > 8).

### 3.2. Microbial Source Tracker in the Sediment and Water Communities

**Figure 1:**
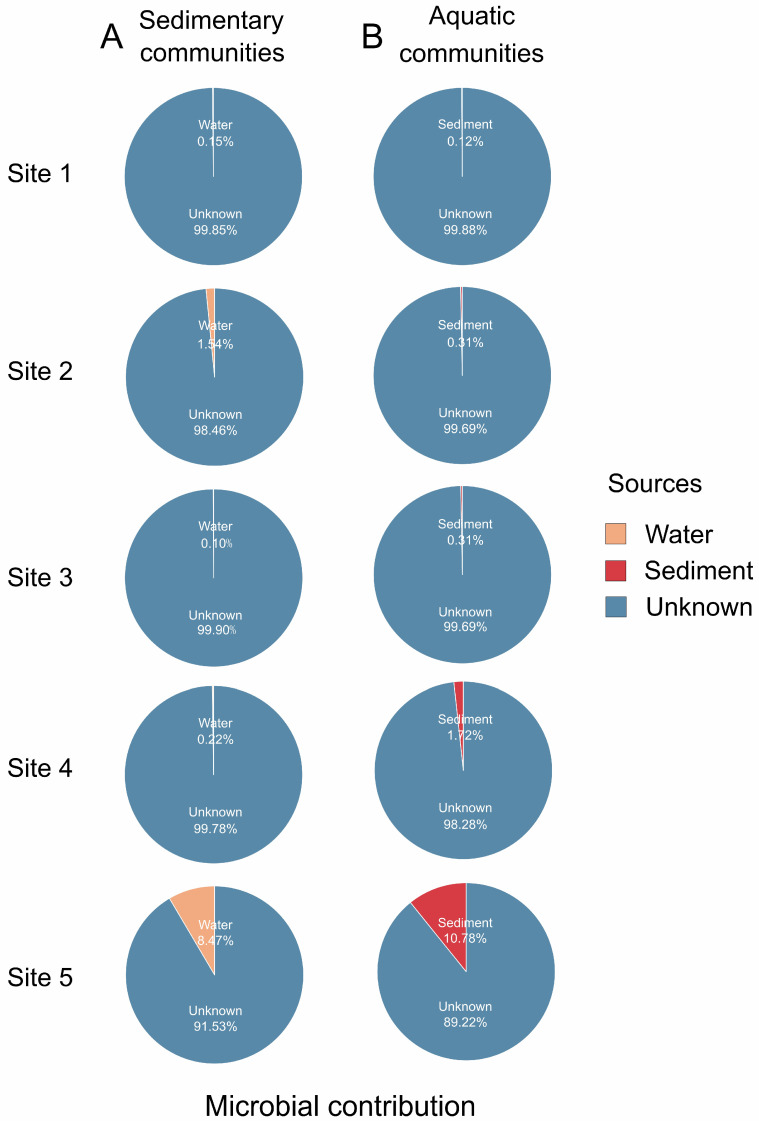
FEAST source tracker analysis showing the contribution of aquatic communities to the lake sediment (**A**) or sedimentary communities to the lake water (**B**) among the five studied sites in the Yuncheng Salt Lake.

The FEAST source tracker analysis showed that the aquatic microbial contribution appeared to be restricted to the corresponding sediment communities, and vice versa. For example, except site 5, only 0.10–1.54% of the total microbial community in lake sediment was found to originate from the corresponding lake water sources. The potential contributing sources were largely unknown (98.46–99.90%) (Figure 1A). Similarly, only 0.12–1.72% of the microbial communities in lake water samples may have originated from corresponding lake sediments, while the significance of the “unknown” fraction (98.28–99.88%) predominated in the lake water samples (Figure 1B). However, at site 5, the microbial contribution in the salt lake sediment and water to each other’s microbial communities increased significantly. Specifically, 8.47% of the microbial communities in the sediments could have originated from lake water, while 10.78% of the sedimentary microorganisms contributed to the aquatic microbial populations (Figure 1).

### 3.3. Diversity and Composition of Microbial Community in the Sediment and Water Samples

A total of 5903 amplicon sequence variants (ASVs) containing 4,161,827 high-quality sequences were obtained in this study. Based on comparisons with the SILVA 138 database, 66 microbial phyla were detected in all samples, including 142 classes, 297 orders, 433 families, and 646 genera. The alpha diversity index was used to compare the differences in richness and diversity of microbial communities (Figure S2). The observed ASVs of the sediment and water samples were 647 ± 331 and 330 ± 93, respectively. The Shannon indices for sediment and water samples were 3.91 ± 1.24 and 2.77 ± 1.01, respectively. The Wilcox test also showed that the observed ASVs and Shannon indices were significantly (*p* < 0.05) higher in sediment samples than in water samples (Figure S2). These results suggest a more diverse microbial community in sediment samples, with a higher number of species observed in lake sediments.

**Figure 2:**
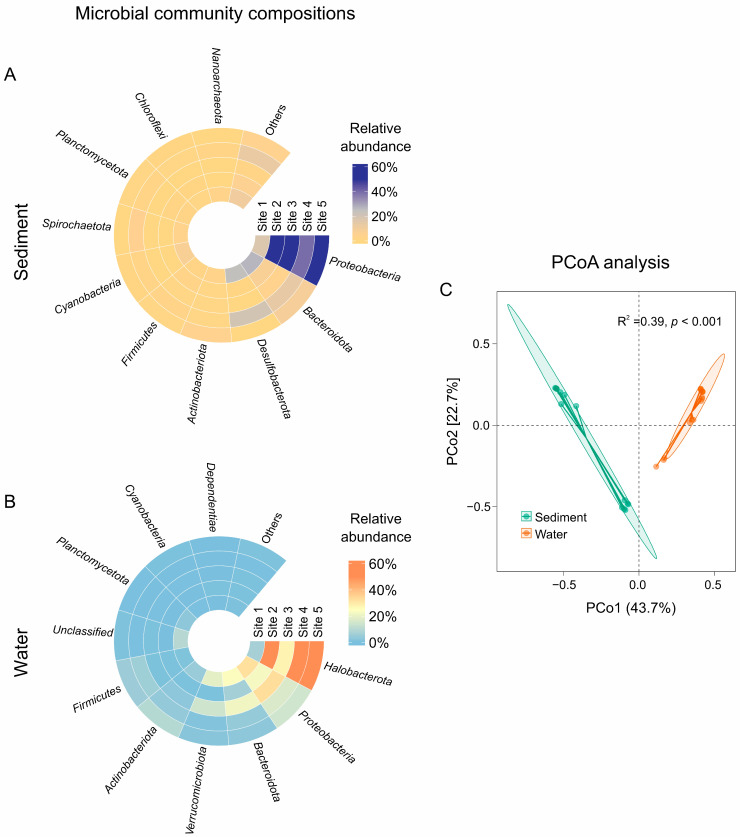
The circus plot depicts the abundance of the microbial community at the phylum level in the sedimentary (**A**) and aquatic (**B**) environments, respectively. The relative values are represented by the color intensity with the legend indicated in the right corner. PCoA analysis based on the Bray-Curtis dissimilarity matrix showing the difference in microbial community composition between the sediment and water samples (**C**). The values on the PCoA axes indicate the percentages of total variation explained by each axis. Microbial community dissimilarities were also indicated by Adonis tests (Bray-Curtis distance, permutations = 999).

**Figure 3:**
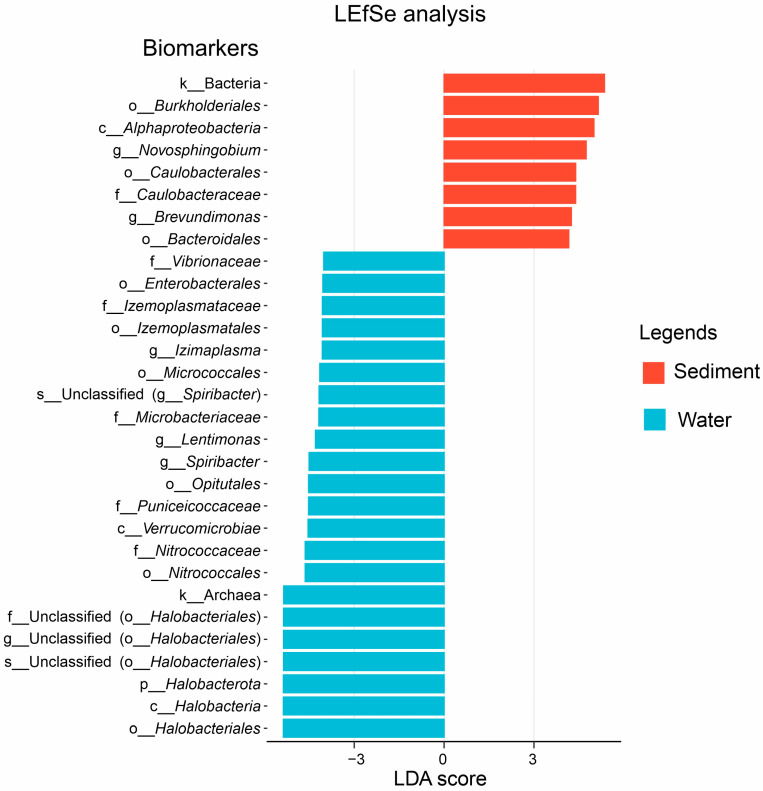
LEfSe analysis of microbial abundance between sediment and water samples. The histogram shows LDA scores calculated for differently abundant microbes (known as “biomarkers”) between sediment and water samples with a threshold of 4.0 (threshold: *p* < 0.01).

Taxonomic assignment showed that the microbial community composition differed between sediment and water samples (Figure 2). At the phylum level, *Proteobacteria*, *Bacteroidota*, and *Desulfobacterota* were the dominant groups in the sediment samples, and their total relative abundance accounted for more than 82.18% (on average) (Figure 2A). *Halobacterota*, *Proteobacteria,* and *Bacteroidota* dominated the microbial communities with relative abundances of 46.94%, 23.74%, and 11.64% of the total microbial population, respectively (Figure 2B). Furthermore, PCoA analysis of microbial communities showed clear separation between the sediment and water samples (Figure 2C). The maximum difference plane in PCoA had >66% explanatory variables (PCo1 43.7%, PCo2 22.7%). The patterns were further confirmed by Adonis, which showed significant (R^2^ = 0.39, *p* < 0.001) habitat effects on microbial community structures. The LEfSe analysis was used to identify the taxonomies causing significant differences between sediment and water samples. The results showed that a total of 30 microbial branches had statistically significant differences (*p* < 0.01) between sediment and water samples with an LDA threshold of 4.0 (Figure 3). The different biomarkers were more abundant in water samples (22 branches) than in sediment samples (8 branches). In particular, members of *Burkholderiales* (order), *Alphaproteobacteria* (class), *Novosphingobium* (genus), *Caulobacterales* (order), and *Bacteroidales* (order) were enriched in sediment samples. While *Halobacteria* (class), *Nitrococcales* (order), *Verrucomicrobiae* (class), *Opitutales* (order), *Micrococcales* (order), *Izemoplasmataceae* (family), and *Enterobacterales* (order) were enriched in water samples.

The composition of microbial communities in lake sediment and water samples was influenced by various environmental factors (Figure S3). Of all the environmental factors examined, the concentrations of SO_4_^2−^ and Ca^2+^ had a strong effect on the microbial community composition in the sediment samples (Mantel test, r = 0.669, *p* = 0.001 and r = 0.213, *p* = 0.038, respectively) (Figure S3A). However, in lake water samples, the microbial community composition was influenced by additional environmental factors including pH, Cl^−^, SO_4_^2−^, Na^+^, K^+^, Mg^2+^, Ca^2+^, and salinity (Mantel test, all *p* < 0.01) (Figure S3B).

### 3.4. Assembly Mechanisms Qualification of Sedimentary and Aquatic Microbial Community

**Figure 4:**
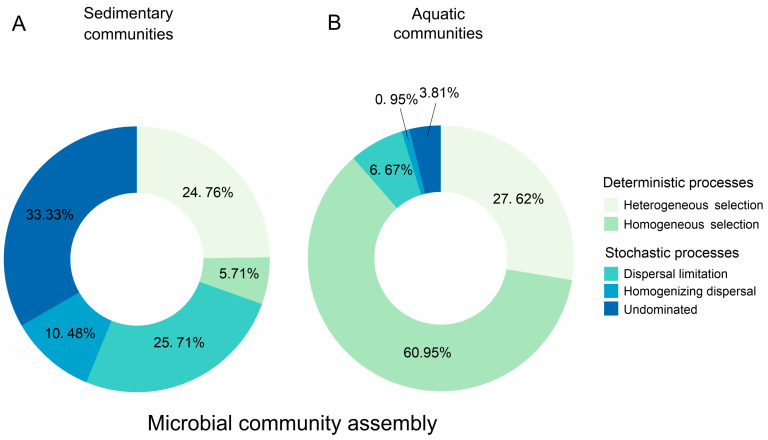
Donut diagrams showing the relative contributions of deterministic and stochastic processes to structure the microbial communities in the studied saline lake sediment (**A**) and water (**B**), respectively.

Distinct deterministic and stochastic processes influenced the compositions of the sedimentary and aquatic microbial community (Figure 4). The microbial community in the studied saline lake sediment samples was predominantly determined by non-dominant processes, dispersal limitation and heterogeneous selection, which explained on average 33.33%, 25.71%, and 24.76% of the community variation, followed by homogenizing dispersal (10.48%) and homogeneous selection (5.71%) (Figure 4A). In contrast, homogeneous selection was the most important process structuring aquatic microbial communities (60.95%), followed by heterogeneous selection (27.62%). Dispersal limitation, non-dominant processes, and homogenizing dispersal played minor roles in structuring aquatic communities, contributing 6.67%, 3.81%, and 0.95% of the variation in lake water samples, respectively (Figure 4B).

### 3.5. Predicted Functional Group Differences Between Sediment and Water Communities

**Figure 5:**
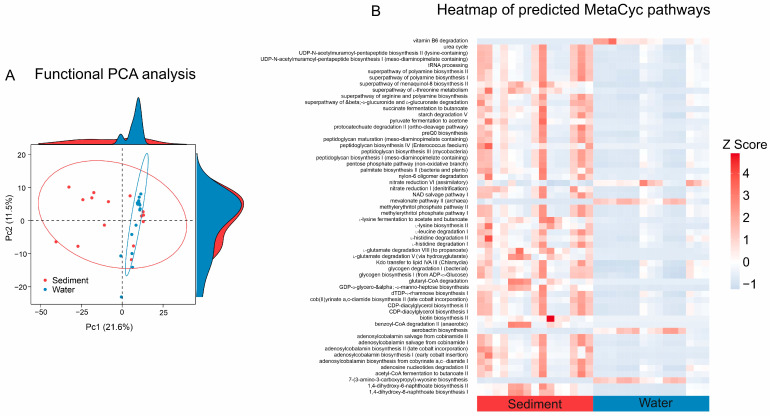
PICRUSt2 analysis of the functional characteristics of the microbial community in the sediment and water samples. (**A**) Principal component analysis showing the differences in MetaCyc pathway signaling between sedimentary and aquatic microbial communities. (**B**) Differential analysis heatmap of predicted MetaCyc pathways of the saline lake sediment and water microbial community.

Functional significance between sediment and water samples was assessed by predicting the metabolic pathways most likely represented by the 16S microbiome profiles. Functional PCA analyses of MetaCyc pathway abundances showed that there was a partial overlap between the microbial functions in the lake sediment and the aquatic community, suggesting shared functionalities. However, most functions within the lake sediment microbial community differed from those in the aquatic microbial community, with the PCA1 axis explaining 21.6% and the PCA2 explaining 11.5% of the variation in abundance (Figure 5A). Picrust2 analysis identified 225 MetaCyc pathways that were statistically different (*p* < 0.05) in sediment samples from those in water samples (Figure 5B). For microbial communities in sediment samples, significant metabolic pathways were mainly involved in the urea cycle, biosynthesis (e.g., pentapeptide, polyamine, lysine, naphthoate, biotin), degradation (e.g., _D_-glucuronate, starch, leucine, histidine, glutamate), fermentation, and dissimilatory nitrate reduction. In the water samples, however, the metabolic pathways with the highest abundance were vitamin B6 degradation, assimilatory nitrate reduction, the mevalonate pathway in archaea, aerobactin, and wyosine biosynthesis. Taken together, our data highlight the potential for significant functional differences between the sediment and water samples.

**Figure 6:**
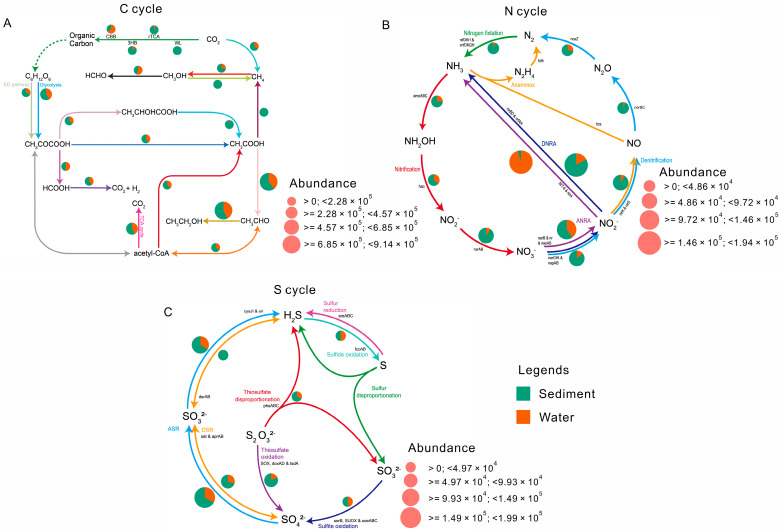
Relative abundance of functional microorganisms involved in the carbon cycle (**A**), nitrogen cycle (**B**), and sulfur cycle (**C**) in saline lake sediment and water samples. The size of pie charts represents the total relative abundance of each pathway. 3HB, 3-Hydroxypropionate Bicycle; CBB, Calvin-Benson-Bassham cycle; TCA, tricarboxylic acid cycle; rTCA, reductive/reverse TCA Cycle; WL, Wood-Ljungdahl pathway; RuBisCo, ribulose-1,5-bisphosphate carboxylase/oxygenase. DNRA, dissimilatory nitrate reduction to ammonia; ANRA, assimilatory nitrate reduction to ammonia. Anammox, anaerobic ammonium oxidation. ASR, assimilatory sulfate reduction; DSR, dissimilatory sulfate reduction.

Differences in the functional microorganisms involved in the process of carbon, nitrogen, and sulfur cycling between saline lake sediment and water samples are shown in Figure 6. In carbon cycling processes, the sediment samples showed a high relative abundance of pathways involved in photosynthetic processes, namely carbon fixation (the 3-hydroxypropionate bicycle, reductive/reverse TCA cycle, and Wood-Ljungdah pathways), central metabolism (Entner-Doudoroff pathway, Gluconeogenesis, and tricarboxylic acid cycle), methane metabolism (methanogenesis and the methane to methanol pathway for methane oxidation), and fermentation (except the pyruvate to formate pathway). In contrast, the water samples had higher relative abundance of the Calvin-Benson-Bassham (CBB) pathway for carbon fixation, the methanol to formaldehyde pathway for methane oxidation, and the pyruvate to formate pathway for fermentation (Figure 6A). Regarding the nitrogen cycle, the abundance of microbes involved in dissimilatory nitrate reduction to ammonia (DNRA), assimilatory nitrate reduction to ammonia (ANRA) (nitrate to nitrite), denitrification, nitrogen fixation, and nitrification pathways was higher in sediment samples than in water samples. However, the water samples had a higher relative abundance of the nitrite to ammonia process in the ANRA pathway (Figure 6B). Regarding the sulfur cycle, microbial assimilatory sulfate reduction (ASR), dissimilatory sulfate reduction (DSR), thiosulfate oxidation, and thiosulfate disproportionation were found to be more common in sediment samples than in water samples. Notably, sulfite and sulfide oxidation showed similar abundances in sediment and water samples.

## 4. Discussion

### 4.1. Contribution of the Lake Water to Microbial Communities in Sediments and Vice Versa

Our data show that despite the frequent exchange between sediment and lake water, very few sediment microorganisms originate from the overlying water, i.e., the contribution of aquatic microbial communities to sediment microbial communities is limited. This finding strongly supports our first hypothesis. The low ability of sediment and water microbial communities to survive in each other’s environments can be attributed to the different physicochemical properties of the two environments. For example, in this study, it was found that the salinity in lake water was significantly higher than that in the sediment (Table S1). In our previous study on hypersaline Lake Chaka, we also observed a decrease in salinity from lake water (325 g/L) to sediment (approximately 40 g/L) [10]. Aquatic microbial communities arise from microorganisms transmitted through precipitation, groundwater, and soil, while sedimentary microbial communities develop over time through accumulation, deposition, and erosion [40]. After frequent exchange, free-living aquatic microbial communities can establish themselves on lake sediments; on the contrary, sediment microbes can also be forced to enter the free-living state in the aquatic environment. This difference in lifestyle likely results in a reduction in microbial survival.

Furthermore, it is noteworthy that the microbial contribution in salt lake sediment and water to each other’s microbial communities increased significantly at site 5 compared to other sampling sites (Figure 1). Such a difference could be attributed to the salinity differences between sediments (with an average salinity of 56.24 g/L) and lake water (96.67 g/L), which at this particular site were not as significant as those observed at other sites (e.g., site 1, sediment 85.63 g/L vs. water 256.47 g/L; site 2, sediment 72.10 g/L vs. water 175.14 g/L; Table S1). In fact, Yuncheng Salt Lake consists of several ponds, each characterized by a unique salinity [41]. Salinity plays a crucial role in shaping the microbial differences between sediments and water columns [9]. Consequently, site 5, which is characterized by a comparatively lower salinity difference between sediments and water, had a microbial source contribution of 8.47%/10.78% to each other’s microbial communities. These results suggest that a reduced salinity gap between sediments and lake water increases the microbial contributions to both sediment and water communities.

Our previous study showed that contributions from onshore soil microorganisms were largely restricted to nearby lake sedimentary and aquatic communities due to habitat heterogeneity and salinity variations [32]. Consequently, it appears that lake sediment, water, and onshore soil do not serve as primary sources for lake-associated microbes. In fact, previous studies have shown that microbial sources in lakes are diverse, including local microbial communities, precipitation, influent water, sediment resuspension, and water mixing [42]. For example, glacial streams are an important source of microorganisms (constituting 45.53% of the bacterial community) in glacial lake water [43]. Furthermore, a study showed that native microbial communities are the most important source of bacteria in lakes [42]. Therefore, it is reasonable to speculate that the microbial assemblages observed in the studied saline lake primarily arise from the cumulative combination of one or more external sources while indicating limited immediate contributions at the community level in the studied sediment and aqueous populations.

### 4.2. Distinct Microbial Community Diversity and Compositions Between Saline Lake Sediment and Water

Microbial diversity often shows strong habitat-specific patterns. In our study, the microbial communities in saline lake sediments showed much higher species richness and Shannon diversity than their counterparts in water. This observation is consistent with global patterns of microbial diversity. This indicates that sediments are more phylogenetically diverse than any other environments [44]. The possible explanation was that more active microbial processes may occur in sediments than in water and that the availability of nutrients also increases [45]. Therefore, the discovery that microbial diversity in sediments is higher than that in water has been reported in various aquatic ecosystems, including lakes [46] and estuaries [45,47]. However, some previous studies have reached the opposite conclusion. Researchers found that taxonomic richness was generally greater in water than that of subseafloor sediments of the deep open ocean [48], or in Arctic marine habitats [49]. These distinct findings can be attributed to habitat heterogeneity: there may be significant differences in microbial biodiversity patterns in oligotrophic and extreme environments such as open oceans and polar regions. According to the differences in microbial diversity, the microbial community structures have significant differences between sediments and water columns in Yuncheng Salt Lake. The high salinity of the Yuncheng Salt Lake could alter the composition, diversity, and interactions within microbial communities, leading to different ecological patterns. For example, in this study, *Alphaproteobacteria* (sediment), *Halobacteria* (water), *Gammaproteobacteria* (water), and *Verrucomicrobiae* (water) were identified as the main contributors to the differences in microbial communities between water and sediment samples. Previous studies have suggested that *Halobacteria* are typical extreme haloalkaliphilic organisms that require a high salinity and alkaline environment to maintain growth and structural stability [50,51]. *Alphaproteobacteria* are generally considered halotolerant groups and can survive in freshwater environments [52,53]. Changes in the composition of microbial communities can result from their adaptation to specific ecological niches [54]. The significant salinity gradients between lake sediment and water can cause physiological limitations on microbial osmoregulation as well as energy and low water availability [55,56,57]. Therefore, salinity exerts a selective pressure on the microbial communities in lake water, resulting in the survival of microorganisms that are better able to adapt to osmotic stress and an increasing variation in microbial composition in the sediment.

Previous studies have also shown that the composition of microbial communities in sediments differs significantly from those in overlying water in less saline environments, including marine environments [58], estuaries [45], and hot springs [59]. For example, the most dominant bacteria were affiliated with *Deltaproteobacteria* and *Gammaproteobacteria* in sediment and with *Alphaproteobacteria* and *Betaproteobacteria* in water in the Yellow River estuary [45]. In contrast to these low-salinity environments, saline lakes are a representative extreme habitat characterized by high salinity and alkaline and various geochemical gradients [60,61], indicating that multiple stressors exist in saline environments. Halophiles that thrive in such harsh saline environments are subject to strong selection pressure compared to less saline environments. In our salt lake habitat, in addition to the *Proteobacteria* succession, we also observed that typical extremely halophilic *Halobacteria* were a distinct group of microorganisms between the sediment and lake water (Figure 2). Therefore, in addition to habitat heterogeneity between sediment and water column, salinity-induced shifts were also crucial for accurate interpretation of the observed microbial dynamics.

### 4.3. Relative Contributions of Deterministic and Stochastic Processes in Shaping the Sedimentary and Aquatic Community Structures

The assembly of sedimentary and aquatic microbial communities follows different patterns, with sedimentary microbial communities being influenced mainly by stochastic processes (non-dominant processes and dispersal limitation) and aquatic microbial communities being controlled predominantly by deterministic processes (homogeneous and heterogeneous selection) (Figure 4). This finding was consistent with previous findings. Due to the high hydraulic conductivity of the aquatic microbial community, their dispersal is generally the least restricted. In contrast, the dispersal of microorganisms in non-liquid sediments should be more strictly restricted [22]. However, previous studies have reported very different mechanisms of community assembly. For example, deterministic processes were reported to dominate the assembly of microbial communities in lake sediments [7,62], while stochastic processes played an important role in explaining lake water diversity patterns [19]. This strong contrast in community assembly mechanisms may be attributed to ecosystem properties that have a significant impact on microbial communities and their assembly processes under environmental perturbations such as trophic state [2,62], temperature fluctuations and microbial interactions [63], salinity [9,64], pH [65], and temporal scales [66]. In this study, the composition of sedimentary microbial communities was less influenced by environmental factors. In contrast, salinity and other environmental variables had a significant impact on the composition of aquatic microbial communities as demonstrated by the Mantel test (Figure S3). Therefore, it is not surprising that stochastic processes play a prominent role in community assembly within saline lake sediments while deterministic processes dominate in saline lake water bodies.

### 4.4. Potential Metabolic Functions of Microbial Players in the Sediment and Water Samples

Microbial communities in lake sediment and water exhibited distinct functional properties and provided compelling evidence for specific habitat patterns. Among the proposed functions, microbial communities in lake sediments showed stronger capabilities in carbon, nitrogen, and sulfur cycling, biosynthesis, and degradation than in lake water (Figure 5 and Figure 6). This difference can be attributed to different chemical and physical environments between sediment and water. Therefore, the processes driving variations in microbial communities also differed between these two environments [5]. This study observed a significant increase in salinity from sediments (salinity increased from 55.20 g/L to 125.20 g/L) to lake water (salinity increased from 96.20 g/L to 296.40 g/L). Salinity is a key factor influencing metabolic functions [67], and most metabolic activities are energy limited under saline and hypersaline conditions [55]. For example, denitrification was relatively more common in sediments than in lake water (Figure 6B). This finding is consistent with a previous study that denitrification was a salt-dependent process and their denitrification rates decreased with increasing salinity [68]. Microorganisms with high salinity require additional energy to cope with osmotic stress [55]. Therefore, it is not surprising that the potential functions of most biogeochemical processes had higher potential in sediments than in lake water. In contrast, certain metabolic pathways remain active even in high-salinity environments; for example, the mevalonate pathway was found to be more abundant in archaea in lake water than in sediments, mainly due to the relatively high abundance of *Halobacteria* archaea in lake water [69]. Furthermore, habitat heterogeneity between sediment and water is another important factor contributing to these functional differences. Previous studies have shown that nitrifying organisms predominantly inhabit sediments; therefore, nitrification is expected to occur primarily within lake sediments [6]. The enrichment of these bacteria in sediments can be attributed to the inhibitory effect of sunlight on nitrification [70], which subsequently reduces nitrification rates in lake water. Therefore, important biogeochemical functions are expected to have a higher potential in lake sediment than in lake water.

### 4.5. Limitations of This Study and Recommendations for Future Microbial Studies in Saline Lakes

Some possible limitations in interpreting our results should be taken into account. First, sampling was conducted in a single period (mid-September), which may not account for seasonal variability. Consequently, the applicability of the observed microbial contributions, community composition, and assembly mechanism differences in sediments and lake water to other seasons remains uncertain. Second, the functional predictions derived from Picrust2, which were based on the existing literature on characterized strains, were relatively conservative. Therefore, the investigation of metabolic functions within the salt lake sediment and water communities remains incomplete in this study. In future studies, a temporal sampling strategy should be implemented to investigate the microbial contributions, community composition, and differences in assembly mechanism between sediment and lake water in saline lakes. In addition, metagenomic sequencing should be used to comprehensively investigate the active metabolic functions within the sediment and water communities of saline lakes.

## 5. Conclusions

In summary, a limited proportion of microbial communities in saline lake sediments can survive in the corresponding waters, and vice versa. However, a smaller salinity gap between sediments and lake water may increase microbial contributions to both sediment and water communities. Habitat heterogeneity and salinity fluctuation resulted in differences in microbial diversity, community composition, and assembly between sediment and water communities. The results of functional predictions showed that sediments have a higher potential for specific biogeochemical processes than overlying waters. These results suggest that frequent exchange could increase the differences in microbial communities, assembly mechanisms, and potential functions between sediment and lake water, and that the microbial contributions depend on the difference in salinity between sediment and water. This study expands our understanding of microbial survival and community assembly mechanisms in sediment and water habitats and lays the foundation for studying how microbial communities in saline lakes cope with the frequent exchanges caused by intensified climate change.

## Data Availability

The data will be made available on a reasonable request.

## Supplementary Materials

The following supporting information can be downloaded at: https://www.mdpi.com/article/10.3390/microorganisms12122534/s1, Figure S1: Location of sampling sites in the Yuncheng Salt Lake, Shanxi Province, China. Red dots indicate sampling sites on the sampling cruise; Figure S2: Differences in the observed ASVs (Amplicon sequence variants) (A) and Shannon indices (B) between sediment and water samples in Yuncheng Salt Lake; Figure S3: Network diagram of correlations between environmental factors and microbial community compositions in sediment (A) and water (B) samples; Table S1: Physicochemical variables of the collected lake sediment and water samples.
