# Supplementary material for: Limited Microbial Contribution in Salt Lake Sediment and Water to Each Other’s Microbial Communities
Source: Microorganisms. 2024 Dec 9;12(12):2534. doi: 10.3390/microorganisms12122534 (PMC11676918; doi:10.3390/microorganisms12122534)

Supplementary materials for

Limited microbial contribution in salt lake sediment and water to each other's microbial communities

Mingxian Han[1], Huiying Yu[2]*, Jianrong Huang[1], Chuanxu Wang[2], Xin Li[2], Xiaodong Wang[1], Liu Xu[1], Jingjing Zhao[1], Hongchen Jiang[1]*

[1]State Key Laboratory of Biogeology and Environmental Geology, China University of Geosciences, Wuhan, 430074, China;

[2]Shanxi Key Laboratory of Yuncheng Salt Lake Ecological Protection and Resource Utilization, College of Life Sciences, Yuncheng University, Yuncheng, 044000, China;

* For correspondence.

Hongchen Jiang

Huiying Yu

E-mail: jiangh@cug.edu.cn

yuhuiying@ycu.edu.cn

Submitted to *Microorganisms*

Nov. 29th, 2024

Contents of this file

Number of pages: 5

Number of figures: 3

Supplementary Figures

Figure S1. Location of sampling sites in the Yuncheng Salt Lake, Shanxi Province, China. Red dots indicate sampling sites on the sampling cruise.

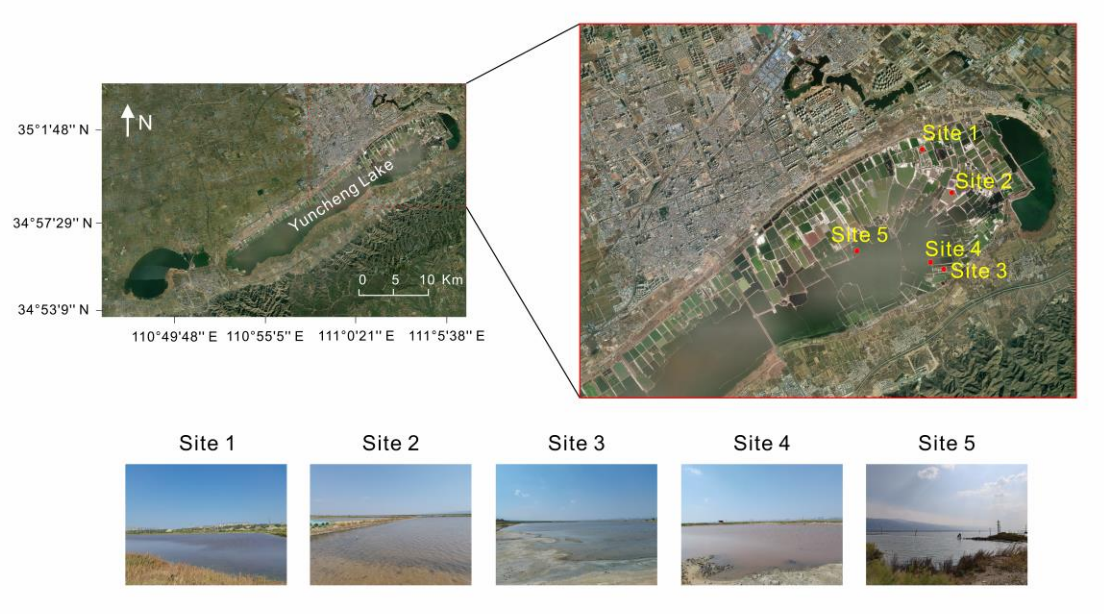

Table S1 Physicochemical variables of the collected lake sediment and water samples

Habitat	Samples	Salinity (g/L)	pH	Cl^- (mg/L)	SO_4^{2-} (mg/L)	Na^+ (mg/L)	K^+ (mg/L)	Mg^{2+} (mg/L)	Ca^{2+} (mg/L)
Sediment	Site1-1	88.00	8.46	25.12	20.06	22.83	0.37	10.11	0.34
	Site1-2	83.80	8.34	25.37	20.56	22.14	0.19	10.16	0.33
	Site1-3	85.10	8.42	25.45	20.62	22.19	0.10	10.10	0.32
	Site2-1	73.00	8.64	27.61	10.12	23.65	0.19	11.48	0.25
	Site2-2	71.90	8.38	27.16	10.35	23.57	0.16	11.43	0.28
	Site2-3	71.40	8.54	27.22	10.33	23.33	0.13	11.54	0.19
	Site3-1	65.33	8.29	24.60	22.04	19.27	0.76	13.61	3.65
	Site3-2	69.10	8.37	24.69	22.65	19.72	0.77	13.79	3.10
	Site3-3	69.40	8.32	24.54	22.63	19.75	0.73	13.68	3.25
	Site4-1	120.67	8.32	4.06	28.68	3.71	0.11	87.05	2.56
	Site4-2	124.20	8.17	4.29	28.60	3.18	0.14	87.14	2.78
	Site4-3	125.20	8.15	4.25	28.64	3.23	0.14	86.11	2.59
	Site5-1	57.33	8.33	1.46	10.01	26.91	0.72	13.10	4.32
	Site5-2	55.20	8.42	1.49	10.19	26.73	0.75	13.90	4.27
	Site5-3	56.20	8.35	1.47	10.15	26.40	0.73	13.43	4.78
Water	Site1-1	256.00	8.34	86.07	8.46	107.19	1.73	51.92	1.83
	Site1-2	256.80	8.24	86.71	8.79	107.18	1.60	51.38	1.60
	Site1-3	256.60	8.30	86.77	8.67	107.17	1.56	50.54	1.57
	Site2-1	179.33	8.48	62.79	7.44	85.56	1.80	37.30	1.71
	Site2-2	172.80	8.34	62.86	7.23	85.86	1.60	37.90	1.56
	Site2-3	173.30	8.44	62.76	7.56	85.98	1.57	37.32	1.53
	Site3-1	201.67	8.69	60.85	6.95	70.62	2.32	44.33	2.09
	Site3-2	201.20	8.43	60.56	7.09	70.85	2.65	44.67	2.67
	Site3-3	200.90	8.66	60.41	6.88	70.52	2.67	44.25	2.65
	Site4-1	294.67	8.24	60.80	13.36	160.50	2.15	65.80	1.17
	Site4-2	295.30	8.51	60.41	13.07	160.65	2.62	65.39	1.31
	Site4-3	296.40	8.35	60.32	13.09	160.32	2.65	65.74	1.45
	Site5-1	97.40	8.75	18.24	4.01	48.15	0.65	19.74	0.35
	Site5-2	96.20	8.58	18.13	4.75	48.45	0.68	17.98	0.61
	Site5-3	96.40	8.66	18.21	4.87	48.90	0.66	17.43	0.59

Figure S2. Differences in the observed ASVs (Amplicon sequence variants) (A) and Shannon indices (B) between sediment and water samples in Yuncheng Salt Lake. Boxes and associated error bars indicate the average observed ASVs and Shannon indices as well as standard deviation in the corresponding sample groups. *: $p < 0.05$, **: $p < 0.01$.

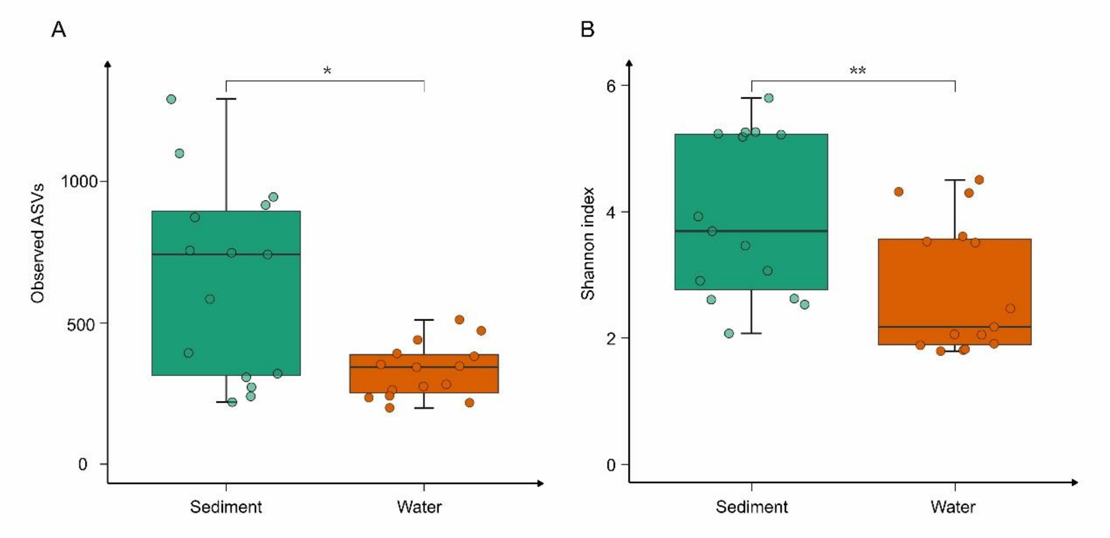

Figure S3. Network diagram of correlations between environmental factors and microbial community compositions in sediment (A) and water (B) samples. Left panel: Mantel test results for the correlation between environmental variables and microbial community composition. Right panel: Spearman's rank correlation (r) of all environmental variables. The edge color indicates Mantel's *p* value based on 9999 permutations.

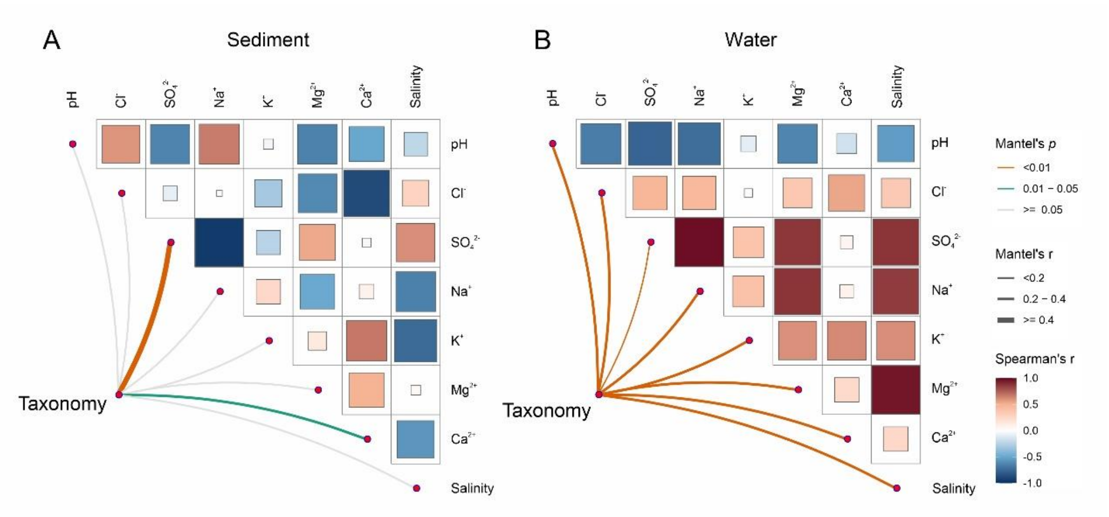

Supplement: Supplementary file 1 [file microorganisms-12-02534-s001.zip › microorganisms-3319277-supplementary.pdf]
